# DO DIFFERENT ADVANCE DIRECTIVES CREATE CONFUSION ON A PATIENT'S WISHES? (MOLST VERSUS FIVE WISHES)

**DOI:** 10.1093/geroni/igac059.2517

**Published:** 2022-12-20

**Authors:** Taylor Perre, Yuchi Young

**Affiliations:** Home Care Association of New York State, Albany, New York, United States; SUNY at Albany School of Public Health, Rensselaer, New York, United States

## Abstract

Introduction. The objective of this study is to compare perspectives of young adults toward advance directives (ADs) and their preferences for life-sustaining treatment and care options. Methods. Participants include graduate students (n=30) attending a New York State university. Data were collected using a structured survey questionnaire, the Medical Orders for Life-Sustaining Treatment (MOLST) form and the Five Wishes form. Summary statistics were performed to address the study aim. Results. Of the participants, the average age was 24 years (60% were female, 60% White, and 27% Black). In Five Wishes, participants who are close to death, 70% wanted all or some forms of life support; when in a coma (47%), or with permanent and severe brain damage (36.6%) chose similar options. In MOLST, without pulse and/or breathing, 87% want CPR; while with pulse and breathing, 96% want artificially administered fluids and nutrition, 90% want mechanical ventilation, 67% want to be hospitalized, 67% want antibiotics, and 53% want unlimited interventions. Conclusion. (1) The majority of participants had not previously completed an AD; however, they were capable of making decisions about their life-sustaining treatments. (2) The discrepancies in treatment preferences may be due to the language of advance directives. Further studies in this respect are warranted.

